# Maternal Pre-Pregnancy Obesity Combined With Abnormal Glucose Metabolism Further Increases Adverse Pregnancy Outcomes in Chinese Pregnant Women

**DOI:** 10.3389/fendo.2021.754406

**Published:** 2022-01-13

**Authors:** Mei-Fang Li, Jiang-Feng Ke, Li Ma, Jun-Wei Wang, Zhi-Hui Zhang, Jing-Bo Li, Lian-Xi Li

**Affiliations:** ^1^ Department of Endocrinology & Metabolism, Shanghai Jiao Tong University Affiliated Sixth People’s Hospital, Shanghai Clinical Medical Center of Diabetes, Shanghai Key Clinical Center of Metabolic Diseases, Shanghai Institute for Diabetes, Shanghai Key Laboratory of Diabetes, Shanghai, China; ^2^ Department of Emergency, Shanghai Jiao Tong University Affiliated Sixth People’s Hospital, Shanghai, China; ^3^ Department of Obstetrics and Gynecology, Shanghai Clinical Center for Severe Maternal Rescue, Shanghai Jiao Tong University Affiliated Sixth People’s Hospital, Shanghai, China; ^4^ Department of Cardiology, Shanghai Jiao Tong University Affiliated Sixth People’s Hospital, Shanghai, China

**Keywords:** pre-pregnancy obesity, gestational abnormal glucose metabolism, gestational diabetes mellitus, adverse pregnancy outcomes, women of reproductive age

## Abstract

**Aims:**

Our aim was to evaluate the separate and combined effects of maternal pre-pregnancy obesity and gestational abnormal glucose metabolism (GAGM) on adverse perinatal outcomes.

**Methods:**

A total of 2,796 Chinese pregnant women with singleton delivery were studied, including 257 women with pre-pregnancy obesity alone, 604 with GAGM alone, 190 with both two conditions, and 1,745 with neither pre-pregnancy obesity nor GAGM as control group. The prevalence and risks of adverse pregnancy outcomes were compared among the four groups.

**Results:**

Compared with the normal group, pregnant women with maternal pre-pregnancy obesity alone, GAGM alone, and both two conditions faced significantly increased risks of pregnancy-induced hypertension (PIH) (odds ratio (OR) 4.045, [95% confidence interval (CI) 2.286–7.156]; 1.993 [1.171–3.393]; 8.495 [4.982–14.485]), preeclampsia (2.649 [1.224–5.735]; 2.129 [1.128–4.017]; 4.643 [2.217–9.727]), cesarean delivery (1.589 [1.212–2.083]; 1.328 [1.095–1.611]; 2.627 [1.908–3.617]), preterm delivery (1.899 [1.205–2.993]; 1.358 [0.937–1.968]; 2.301 [1.423–3.720]), macrosomia (2.449 [1.517–3.954]; 1.966 [1.356–2.851]; 4.576 [2.895–7.233]), and total adverse maternal outcomes (1.762 [1.331–2.332]; 1.365 [1.122–1.659]; 3.228 [2.272–4.587]) and neonatal outcomes (1.951 [1.361–2.798]; 1.547 [1.170–2.046]; 3.557 [2.471–5.122]). Most importantly, there were no obvious risk differences in adverse pregnancy outcomes between maternal pre-pregnancy obesity and GAGM group except PIH, but pregnant women with both obesity and GAGM exhibited dramatically higher risks of adverse pregnancy outcomes than those with each condition alone.

**Conclusions:**

Maternal pre-pregnancy obesity and GAGM were independently associated with increased risks of adverse pregnancy outcomes. The combination of pre-pregnancy obesity and GAGM further worsens adverse pregnancy outcomes compared with each condition alone.

## Introduction

Over the past decades, the incidence of abnormal glucose metabolism (AGM) is increasing together with maternal obesity, especially for women of reproductive age (1, 2). Similar trends emerge in China after the announcement on China’s universal two-child policy since 2015; an ever-increasing number of women with pre-pregnancy obesity and gestational AGM (GAGM) gave birth to a sheer number of infants (3, 4). As a result, more and more studies began to focus on the effects of maternal pre-pregnancy obesity or GAGM on adverse maternal and neonatal outcomes (5–7). For example, Athukorala et al. (6) demonstrated that obese women faced an elevated risk of developing gestational diabetes mellitus (GDM), pregnancy-induced hypertension (PIH), and pre-eclampsia among the Australian obstetric population. A cross-sectional study conducted by Billionnet et al. (7) also showed that GDM was linked to a moderately rising risk of adverse perinatal outcomes from 716,152 births in France.

However, GAGM is often accompanied with obesity, and studies regarding the comparisons between the influence of maternal pre-pregnancy obesity without GAGM and maternal GAGM without pre-pregnancy obesity on adverse perinatal outcomes are very limited. Furthermore, whether maternal pre-pregnancy obesity combined with GAGM has further influences on adverse pregnancy outcomes remains unclear among Chinese pregnant women.

Therefore, we conducted this retrospective cohort study to assess the significance of maternal pre-pregnancy obesity, GAGM alone, and combined above conditions on a spectrum of adverse maternal and offspring pregnancy outcomes among Chinese pregnant women. In addition, we further compared the maternal and neonatal outcomes between maternal pregnancy obesity and GAGM alone to further clarify which condition was worse.

## Materials and Methods

### Study Population

This retrospective cohort study was based on data from our previous studies (8, 9). In brief, 4,178 singleton pregnancies who underwent an oral glucose tolerance test (OGTT) during 24–28 weeks and delivered in the obstetrics department of Shanghai Jiao Tong University Affiliated Sixth People’s Hospital were continuously recruited during the period from January to December in 2016. Among them, 1,382 subjects were excluded as follows: lack of information on demographics, delivery, maternal and neonatal outcomes, and OGTT in our hospital; pre-pregnancy BMI (ppBMI) < 18.5 kg/m^2^; reproductive system anatomy abnormalities; and a prior history of diabetes, chronic diseases, and malignancy. Ultimately, 2,796 subjects were enrolled in the present study.

To investigate the independent and joint effects of maternal pre-pregnancy obesity and GAGM on the pregnancy outcomes, all the objects were stratified into four groups by the results of ppBMI and OGTT, including normal group (neither pre-pregnancy obesity nor GAGM), women with pre-pregnancy obesity but without GAGM (pre-pregnancy obesity alone group), women with GAGM but without pre-pregnancy obesity (GAGM alone group), and women with both two conditions (pre-pregnancy obesity and GAGM group).

Our study was approved by the Human Research and Ethic Committee of Shanghai Jiao Tong University Affiliated Sixth People’s Hospital and adhered to the tenets of the Declaration of Helsinki, and written informed consents were obtained from all participants.

### Data Collection and OGTT

Data of maternal age, height and body weight before pregnancy (self-reported), primiparity, number of previous pregnancies and births, gestational age, gestational weight gain, gender of newborn, birth weight and height and Apgar scores, and pregnancy outcomes from the participants’ hospital admission/discharge records were collected. PpBMI (kg/m^2^) was measured as an individual’s weight (kg) divided by the square of height (m).

The standard OGTT was conducted after an 8- to 10-h fasting at 24 to 28 weeks of gestation. 75 g glucose was administered orally within 5 min, and the blood samples of pregnant women were collected for measuring blood glucose (BG), insulin, glycosylated hemoglobin A1c (HbA1c), and glycated albumin (GA) before and after taking the glucose.

### Diagnostic Criteria and Outcomes

PpBMI was classified based on the Asia-Pacific criteria set by WHO as underweight (ppBMI < 18.5 kg/m^2^), normal weight (18.5 ≦̸ ppBMI < 23 kg/m^2^), overweight (23 ≦̸ ppBMI < 25 kg/m^2^), and obese (ppBMI ≧̸25 kg/m^2^) (10). GAGM consisted of GDM and pregnancy with diabetes (PWD). According to the standard of International Association of Diabetes and Pregnancy Study Groups (IADPSG) (11), GDM was defined as fasting blood glucose (FBG) at least 5.1 mmol/l during the first trimester or when one of the 75-g OGTT conditions met the following: FBG at least 5.1 mmol/l; 1 h after glucose load at least 10.0 mmol/l; and 2 h after glucose load at least 8.5 mmol/l. PWD was defined as FBG at least 7.00 mmol/l or HbA1c at least 6.5% or randomized BG at least 11.1 mmol/l when first found either on the very first prenatal examination or on the OGTT.

As depicted in our recent studies (8, 9), adverse maternal outcomes consisted of PIH (defined as new-onset systolic blood pressure ≧̸140 mmHg and/or diastolic blood pressure ≧̸90 mmHg occurring after 20 weeks’ gestation), preeclampsia (defined as PIH combined with proteinuria 0.3 g/24 h), preterm delivery (the delivery < 37 weeks’ gestation), cesarean delivery (CS, recorded by the recorded on the delivery by a midwife or doctor), postpartum hemorrhage (blood loss ≧̸500 ml within the first 24 h of delivery), placental previa (the placenta grew in the lowest part of the uterus and covered all or part of the opening to the cervix), and abruption (the normally located placenta separated from the uterine site before the birth of fetus), as well as dystocia (abnormal or difficult childbirth or labor diagnosed by an obstetrician). Total adverse maternal outcomes were referred as one or more of the above complications. Adverse neonatal outcomes consisted of low birth weight (birth weight < 2,500 g), macrosomia (birth weight ≧̸4,000 g), fetal asphyxia (identified when Apgar score < 7 at 1 min), fetal distress (defined as when the symptoms of fetal hypoxia occurred, including fetal bradycardia, serious variable decelerations and lasting late decelerations), deformities (diagnosed either by ultrasonography in pregnancy or during the neonatal period), and stillbirth (referred as a baby born with no signs of life with gestation period of ≧̸28 weeks). Total adverse neonatal outcomes were referred as one or more of the above complications.

### Statistical Analyses

All statistical analyses were performed by SPSS 19.0. Mean ± standard deviation or median (interquartile range) or frequency (percent) was used to present descriptive data. Statistical differences among the group comparisons were tested using one-way ANOVA with LSD for continuous variables with normal distribution, Kruskal–Wallis test for continuous variables with non-normal distribution, and chi-squared analysis for categorical variables, respectively. Multiple logistic regression analysis was used to evaluate the independent and combined influences of maternal pre-pregnancy obesity and GAGM on adverse pregnancy outcomes in pregnant women with maternal age, gestational age, and primiparity included as covariates to control for confounding factors. Non-normally distributed variables such as maternal age and gestational age were naturally log-transformed into approximately normally distributed data before regression analysis was conducted. p < 0.05 was considered statistically significant.

## Results

### Clinical and Biochemical Characteristics of the Studied Subjects

Of all 2,796 enrolled women, the mean age of mothers was 30.0 years and the mean ppBMI was 22.31 kg/m^2^. The demographic and clinical characteristics of maternal and their offspring according to maternal pre-pregnancy obesity and GAGM are shown in **Table 1**. Compared with the normal group, maternal pre-pregnancy obesity and GAGM alone and two combined groups have significantly higher maternal age, ppBMI, number of previous pregnancies, related indicators of 75 g OGTT including FBG, 1 h postprandial plasma glucose (PPG), 2 h PPG, fasting insulin, 1 h insulin, 2 h insulin, HbA1c, neonatal birth weight and lower primiparity, gestational weight gain, and Apgar scores. In addition, number of previous births and GA were significantly different among these four groups even after maternal age was adjusted (all p < 0.05).

**Table 1:** Characteristics of pregnant women and their neonates according to maternal pre-pregnancy obesity and GAGM.

Variables	Normal (n = 1,745)	Obese alone (n = 257)	GAGM alone (n = 604)	Obese with GAGM (n = 190)	p value	p’ value
*Maternal characteristics*						
Maternal age (years)*^a^*	29.0 (27.0–32.0)	30.0 (27.0–34.0)	30.0 (27.0–34.0)	32.0 (29.0–35.0)	<0.001	
Gestational age (weeks)*^a^*	39 (38–40)	39 (38–40)	39 (38–40)	39 (38–40)	0.002	0.099
Pre-pregnancy BMI (kg/m^2^)*^a^*	20.83 (19.71–22.48)	26.56 (25.59–28.04)	21.48 (20.07–23.01)	27.34 (25.78–30.05)	<0.001	<0.001
Primiparity, n (%)	1,099 (63)	136 (52.9)	342 (56.6)	91 (47.9)	<0.001	<0.001
Number of previous pregnancies, n (%)					<0.001	<0.001
0 time	810 (46.4)	106 (41.2)	240 (39.7)	63 (33.2)		
1 times	481 (27.6)	58 (22.6)	178 (29.5)	56 (29.5)		
2 times and above	454 (26)	93 (36.2)	186 (30.8)	71 (37.3)		
Number of previous births, n (%)					<0.001	<0.001
0 time	1,099 (63)	136 (52.9)	342 (56.6)	91 (47.9)		
1 times	591 (34)	114 (44.4)	226 (37.4)	93 (48.9)		
2 times and above	53 (3.0)	7 (2.7)	36 (6)	6 (3.2)		
gestational weight gain (kg)*^a^*	13 (10–15)	10.5 (8–14)	12 (10–15)	10 (7–14)	<0.001	<0.001
75 g OGTT						
FPG (mmol/l)*^a^*	4.20 (4.02–4.42)	4.28 (4.13–4.51)	4.44 (4.23–4.73)	4.51 (4.34–4.95)	<0.001	<0.001
1 h PPG (mmol/l)*^a^*	7.41 (6.52–8.38)	7.83 (6.71–8.47)	9.07 (7.50–10.12)	8.86 (7.15–10.22)	<0.001	<0.001
2 h PPG (mmol/l)*^a^*	6.14 (5.29–6.92)	6.10 (4.96–6.97)	7.57 (6.06–8.77)	6.99 (5.50–8.69)	<0.001	<0.001
FIN (mU/l)*^a^*	7.94 (5.81–10.82)	11.22 (7.95–14.81)	8.68 (6.54–12.24)	13 (9.89–17.22)	<0.001	<0.001
1 h IN (mU/l)*^a^*	67.4 (46.1–92.05)	83.90 (57.50–120.5)	70 (48.25–101)	85.8 (56.75–106.5)	<0.001	<0.001
2 h IN (mU/l)*^a^*	53.16 (29.68–81.93)	61.92 (40.37–101.03)	69.69 (45.28–102.93)	71.77 (39.82–112.15)	<0.001	<0.001
HbA1c (%)*^a^*	4.9 (4.7–5.1)	5 (4.7–5.1)	5.0 (4.8–5.2)	5.1 (4.9–5.4)	<0.001	<0.001
GA (%)*^a^*	12.1 (11.6–12.8)	11.9 (11.3–12.6)	12.4 (11.6–13.3)	12.1 (11.1–13.1)	<0.001	<0.001
*Newborn characteristics*						
Male, n (%)	909 (52.1)	118 (45.9)	311 (51.5)	104 (54.7)	0.236	0.988
Birth weight (g)*^a^*	3,320 (3070–3580)	3,390 (3,113–3,675)	3,380 (3,070–3,670)	3,430 (3,135–3,813)	<0.001	0.001
Birth height (cm)	49.62 ± 2.57	49.58 ± 2.64	49.58 ± 2.81	49.59 ± 4.10	0.993	0.985
Apgar scores	9.91 ± 0.87	9.88 ± 0.96	9.84 ± 0.95	9.75 ± 0.93	0.145	0.039

GAGM, gestational abnormal glucose metabolism; BMI, body mass index; OGTT, oral glucose tolerance test; FPG, fasting plasma glucose; 1 h PPG, 1 h postprandial plasma glucose; 2 h PPG, 2 h postprandial plasma glucose; FIN, fasting insulin; 1 h IN, 1 h insulin; 2 h IN, 2 h insulin; HbA1c, glycosylated hemoglobin A1c; GA, glycated albumin. Continuous variables were expressed as mean ± standard deviation or median with interquartile range, while categorical variables were expressed as percentages.

^a^Non-normal distribution of continuous variables. p’ value: adjusted for maternal age.

### Associations of Maternal Pre-Pregnancy Obesity and GAGM and Adverse Maternal Outcomes in Chinese Pregnant Women

The associations of maternal pre-pregnancy obesity and GAGM with adverse maternal outcomes are presented in **Table 2**. Compared with the normal group, other three groups have remarkably increased risks for PIH, preeclampsia, CS, preterm delivery, and total adverse maternal outcomes even after multivariable adjustments. In addition, successively compared with the pre-pregnancy obesity alone and GAGM alone groups, pregnancies with both two conditions have also markedly higher risks for PIH, CS, and total adverse maternal outcomes. Interestingly, pregnancies with both two conditions were observed to have obviously higher risks of preeclampsia and preterm delivery in relation to the GAGM group, but not to pre-pregnancy obesity alone. However, no statistical significance was found on the risks of postpartum hemorrhage, placental previa, and abruption, as well as dystocia among the four groups.

**Table 2:** Association of maternal pre-pregnancy obesity and GAGM and adverse maternal outcomes.

Variables	Normal	Obese alone	GAGM alone	Obese with GAGM	p value
PIH, n (%)	34 (1.9)	21 (8.2)	25 (4.1)	30 (15.8)	
Unadjusted OR (95% CI)	1 (ref)	4.478 (2.556–7.845)^*^	2.173 (1.285–3.673)^*^	9.436 (5.627–15.823)	<0.001
Adjusted OR (95% CI)	1 (ref)	4.045 (2.286–7.156)^*^	1.993 (1.171–3.393)^*^	8.495 (4.982–14.485)	<0.001
Preeclampsia, n (%)	23 (1.3)	10 (3.9)	18 (3)	12 (6.3)	
Unadjusted OR (95% CI)	1 (ref)	3.031 (1.426–6.445)	2.300 (1.232–4.292)^*^	5.047 (2.470–10.316)	<0.001
Adjusted OR (95% CI)	1 (ref)	2.649 (1.224–5.735)	2.129 (1.128–4.017)^*^	4.643 (2.217–9.727)	<0.001
CS, n (%)	634 (36.3)	125 (48.6)	274 (45.4)	121 (63.7)	
Unadjusted OR (95% CI)	1 (ref)	1.659 (1.275–2.159)^*^	1.455 (1.206–1.755)^*^	3.073 (2.251–4.195)	<0.001
Adjusted OR (95% CI)	1 (ref)	1.589 (1.212–2.083)^*^	1.328 (1.095–1.611)^*^	2.627 (1.908–3.617)	<0.001
Preterm delivery, n (%)	93 (5.3)	27 (10.5)	44 (7.3)	25 (13.2)	
Unadjusted OR (95% CI)	1 (ref)	2.085 (1.329–3.271)	1.396 (0.963–2.023)^*^	2.691 (1.683–4.305)	<0.001
Adjusted OR*^a^* (95% CI)	1 (ref)	1.899 (1.205–2.993)	1.358 (0.937–1.968)^*^	2.301 (1.423–3.720)	0.001
Postpartum hemorrhage, n (%)	38 (2.2)	7 (2.7)	10 (1.6)	6 (3.0)	
Unadjusted OR (95% CI)	1 (ref)	1.258 (0.556–2.847)	0.756 (0.374–1.527)	1.465 (0.611–3.512)	0.582
Adjusted OR (95% CI)	1 (ref)	1.254 (0.552–2.848)	0.739 (0.364–1.500)	1.405 (0.579–3.412)	0.591
Placenta previa, n (%)	22 (1.3)	4 (1.6)	11 (1.8)	6 (3.2)	
Unadjusted OR (95% CI)	1 (ref)	1.238 (0.423–3.623)	1.453 (0.700–3.014)	2.554 (1.022–6.379)	0.233
Adjusted OR (95% CI)	1 (ref)	0.938 (0.314–2.799)	1.231 (0.586–2.589)	1.877 (0.731–4.819)	0.590
Placenta abruption, n (%)	2 (0.1)	1 (0.4)	0 (0)	0 (0)	
Unadjusted OR (95% CI)	1 (ref)	3.404 (0.308–37.678)	–	–	0.802
Adjusted OR (95% CI)	1 (ref)	3.199 (0.277–36.965)	–	–	0.833
Dystocia, n (%)	39 (2.2)	2 (0.8)	11 (1.8)	4 (2.1)	
Unadjusted OR (95% CI)	1 (ref)	0.343 (0.082–1.429)	0.811 (0.413–1.595)	0.941 (0.332–2.662)	0.500
Adjusted OR (95% CI)	1 (ref)	0.404 (0.096–1.688)	0.925 (0.467–1.832)	1.248 (0.435–3.586)	
Total adverse maternal outcomes, n (%)	741 (42.5)	153 (59.5)	321 (53.1)	140 (73.4)	
Unadjusted OR (95% CI)	1 (ref)	1.993 (1.527–2.602)^*^	1.537 (1.276–1.851)^*^	3.794 (2.710–5.312)	<0.001
Adjusted OR (95% CI)	1 (ref)	1.762 (1.331–2.332)^*^	1.365 (1.122–1.659)^*^	3.228 (2.272–4.587)	<0.001

Adjusted OR: adjusted for maternal age, gestational age, and primiparity. Adjusted OR^a^: adjusted for maternal age, and primiparity. ^*^p < 0.05 vs. obese with GAGM.

### Association of Maternal Pre-Pregnancy Obesity and GAGM and Adverse Neonatal Outcomes in Chinese Pregnant Women

**Table 3** displays adverse neonatal outcomes resulted from maternal pre-pregnancy obesity and GAGM. Compared with being non-obesity and normal glycometabolism, being pre-pregnancy obesity alone, being GAGM alone, and being two conditions have significantly increased risks of macrosomia and total adverse neonatal outcomes, respectively. In addition, pre-pregnancy obesity combined with GAGM pregnancies has dramatically elevated risks of macrosomia and total adverse neonatal outcomes compared with pre-pregnancy obesity alone and being GAGM alone, respectively. Moreover, it was found that only pregnancies with both pre-pregnancy obesity and GAGM faced prominently higher risks of lower birth weight and fetal asphyxia when compared with healthy pregnancies. However, the risks of fetal distress, fetal deformities, and stillbirth did not exhibit statistical significance among the four groups.

**Table 3:** Association of maternal pre-pregnancy obesity and GAGM and adverse neonatal outcomes.

Variables	Normal	Obese alone	GAGM alone	Obese with GAGM	p value
Low birth weight, n (%)	53 (3.0)	12 (4.7)	28 (4.6)	15 (7.9)	
Unadjusted OR (95% CI)	1 (ref)	1.564 (0.824–2.968)	1.552 (0.972–2.477)	2.736 (1.511–4.956)	0.006
Adjusted OR (95% CI)	1 (ref)	0.999 (0.460–2.169)^*^	1.429 (0.837–2.438)	2.539 (1.295–4.981)	0.045
Macrosomia, n (%)	80 (4.6)	25 (9.7)	51 (8.4)	33 (17.4)	
Unadjusted OR (95% CI)	1 (ref)	2.243 (1.402–3.587)^*^	1.919 (1.334–2.763)^*^	4.375 (2.825–6.774)	<0.001
Adjusted OR (95% CI)	1 (ref)	2.449 (1.517–3.954)^*^	1.966 (1.356–2.851)^*^	4.576 (2.895–7.233)	<0.001
Fetal asphyxia, n (%)	17 (1.0)	4 (1.6)	11 (1.8)	5 (2.6)	
Unadjusted OR (95% CI)	1 (ref)	1.607 (0.536–4.814)	1.886 (0.878–4.048)	2.747 (1.002–7.532)	0.165
Adjusted OR (95% CI)	1 (ref)	0.808 (0.207–3.147)	1.754 (0.695–4.426)	2.966 (1.303–9.740)	0.230
Fetal distress, n (%)	43 (2.5)	9 (3.5)	9 (1.5)	4 (2.1)	
Unadjusted OR (95% CI)	1 (ref)	1.436 (0.692–2.983)	0.599 (0.290–1.236)	0.851 (0.302–2.398)	0.320
Adjusted OR (95% CI)	1 (ref)	1.633 (0.781–3.413)	0.666 (0.321–1.382)	1.069 (0.375–3.047)	0.318
Fetal deformities, n (%)	14 (0.8)	6 (2.3)	8(1.3)	1 (0.5)	
Unadjusted OR (95% CI)	1 (ref)	0.375 (0.037–3.795)	1.660 (0.693–3.976)	1.660 (0.693–3.976)	0.130
Adjusted OR (95% CI)	1 (ref)	1.835 (0.565–5.964)	1.036 (0.336–3.195)	0.375 (0.037–3.795)	0.570
Stillbirth, n (%)	7 (0.4)	3 (1.2)	5 (0.8)	2 (1.0)	
Unadjusted OR (95% CI)	1 (ref)	2.933 (0.753–11.413)	1.655 (0.483–5.674)	2.641 (0.545–12.806)	0.373
Adjusted OR (95% CI)	1 (ref)	1.489 (0.300–7.390)	1.130 (0.279–4.582)	2.078 (0.348–12.394)	0.860
Total adverse neonatal outcomes, n (%)	182 (10.4)	51 (19.8)	95 (15.7)	56 (29.5)	
Unadjusted OR (95% CI)	1 (ref)	2.216 (1.509–2.996)^*^	1.603 (1.227–2.094)^*^	3.589 (2.535–5.018)	<0.001
Adjusted OR (95% CI)	1 (ref)	1.951 (1.361–2.798)^*^	1.547 (1.170–2.046)^*^	3.557 (2.471–5.122)	<0.001

Adjusted OR: adjusted for maternal age, gestational age, and primiparity.

^*^p < 0.05 vs. obese with GAGM.

### Comparisons of Adverse Maternal and Neonatal Outcomes Between Pre-Pregnancy Obesity Alone and GAGM Alone

**Figure 1:**
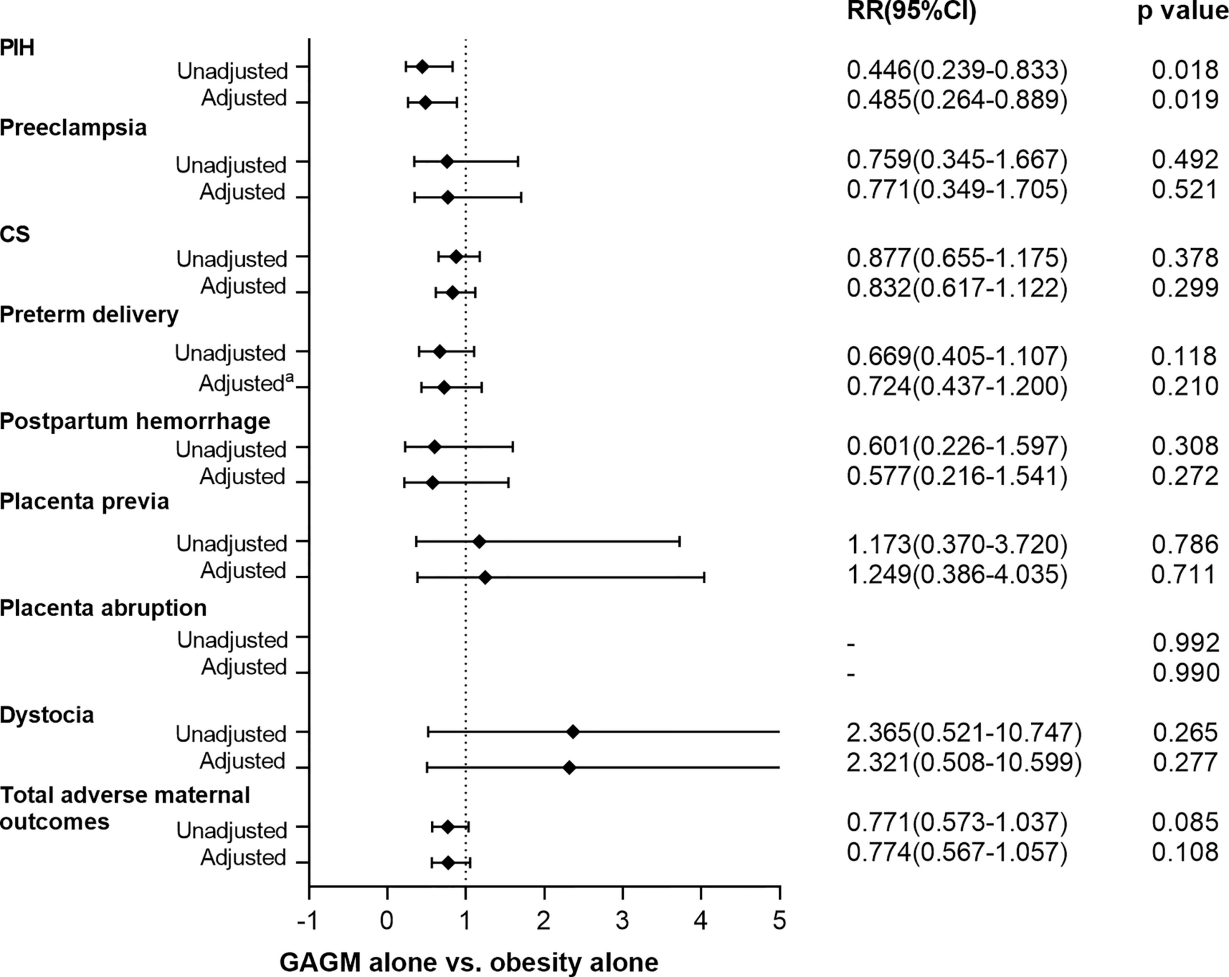
Comparison of adverse maternal outcomes between pre-pregnancy obesity alone and GAGM alone. Adjusted: adjusted for maternal age, gestational age, and primiparity. Adjusted^a^: adjusted for maternal age, and primiparity.

**Figure 2:**
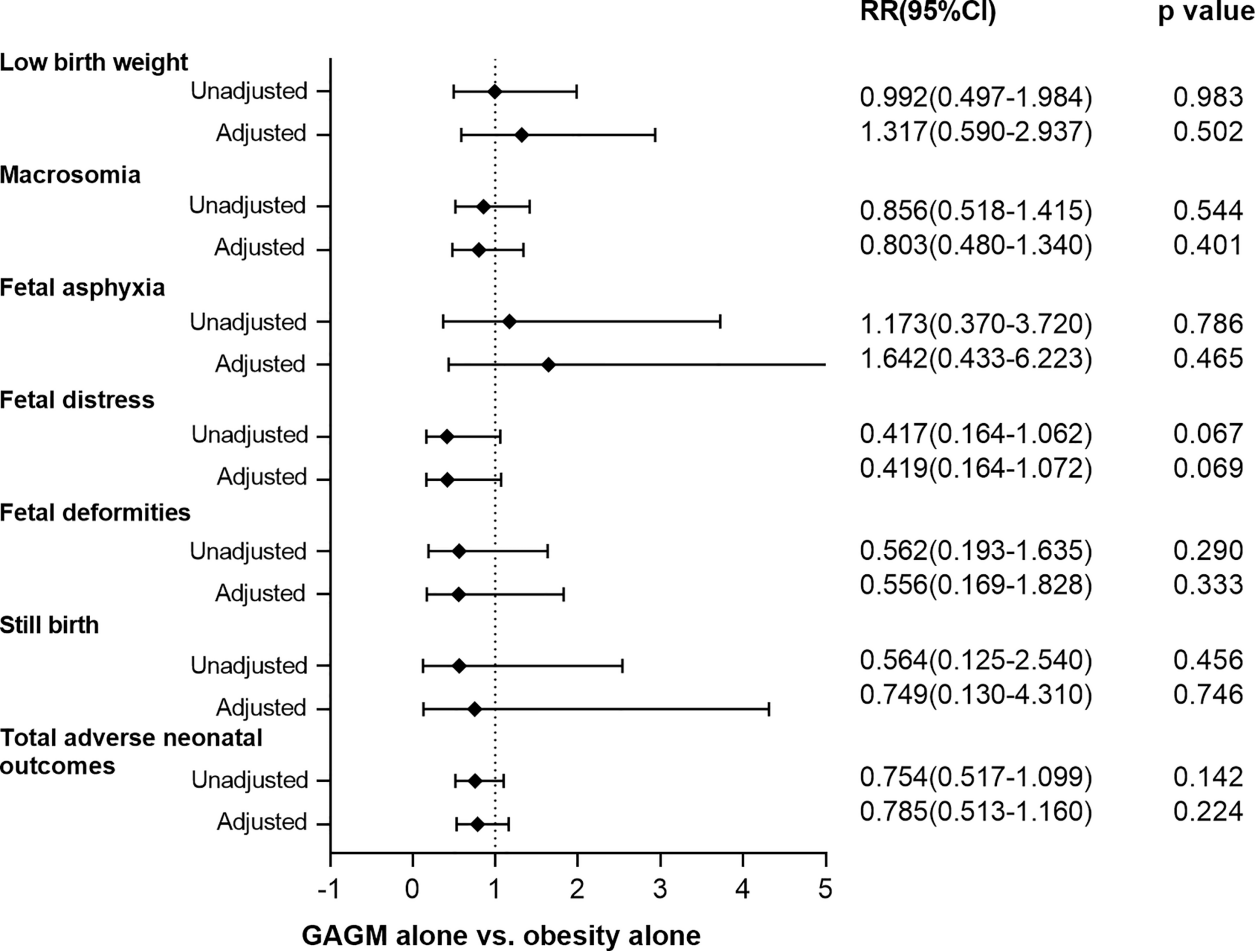
Comparison of adverse neonatal outcomes between pre-pregnancy obesity alone and GAGM alone. Adjusted: adjusted for maternal age, gestational age, and primiparity. Adjusted^a^: adjusted for maternal age, and primiparity.

**Figures 1**, **2** display the comparisons of adverse maternal and neonatal outcomes between pre-pregnancy obesity alone and GAGM alone. Only significantly decreased risk of PIH in the GAGM group was found when compared with pre-pregnancy obesity alone, and no significant differences of other adverse outcomes were observed between two groups.

## Discussion

In the present study, we found that maternal pre-pregnancy obesity and GAGM—both alone and combined—remarkably increased the risks of PIH, preeclampsia, CS, preterm delivery, macrosomia, and total adverse maternal outcomes and neonatal outcomes even after adjustment for confounders, and the maternal and neonatal outcomes for pregnant women with both pre-pregnancy obesity and GAGM were worse than those with only one condition.

For maternal obesity without GDM, most studies mainly focus on its effects on neonatal macrosomia and CS. A prospective cohort study (12) displayed that obese women had 5.42-fold higher odds of having a macrosomic infant than non-obese women when they adopted women without GDM as their studied objects. A register-based study by Ijäs et al. (13) also demonstrated a 1.5-fold risk for macrosomia and 1.52-fold risk for CS in obese mothers without GDM in relation to normal weight mothers without GDM. Consistent with them, our study revealed that the maternal obesity alone group had a 2.449-fold risk of macrosomia and 1.589-fold risk of CS compared with the maternal non-obese group without GAGM. Recently, two population-based cohort studies from Washington and Sweden reported that maternal obesity had higher risk of severe pregnancy outcomes such as malformations and stillbirth (14, 15). In contrast, no significant risk difference of abovementioned adverse pregnancy outcomes was found in normal GAGM women with or without obesity in our present study. This discrepancy may mainly result from the rapid growth in the category of BMI 40–50 kg/m^2^ in the developed countries (16, 17), whereas BMI more than 40 kg/m^2^ was extremely scarce in Asian women. Additionally, the very low rate of serious perinatal outcomes in our present study thanks to the successful establishment and implementation of maternity obstetric emergency cares in Shanghai (18) may also help to explain the above conflicting results.

For GDM women without obesity, whether GDM influences on adverse pregnancy outcomes remains controversial. For example, contrary to our study, the studies by Ijäs et al. (13) and Wahabi et al. (19) displayed that a nearly equal risk of macrosomia and CS was found in normal weight women with and without GDM. The difference between us could be explained by the fact that about 60% GDM women in our study implemented doctors’ advice while all GDM women in Ijäs et al. and Wahabi et al. (19) received dietary counseling, began self-monitoring on their blood glucose, and adopted insulin treatment if their blood glucose repeatedly exceeded the target glucose concentrations. Moreover, a randomized controlled trial by Crowther et al. (20) and our previous study (8), which revealed that the treatment of mild GDM decreased risks of macrosomia, CS, and preeclampsia, also clarified the difference in results. The above studies indicated that nutritional therapy and counseling, blood glucose self-monitoring, and routine follow-up for GDM should be popularized to improve outcomes in clinical practice.

Recently, the concomitant effects of GDM and maternal obesity on perinatal outcomes aroused great interests from scholars. The Hyperglycemia and Adverse Pregnancy Outcome (HAPO) study demonstrated that maternal GDM alone and obesity alone were associated with increased risks of preeclampsia, CS, and macrosomia, and their combination had a greater impact than either one alone; however, the maternal BMI in their study was determined at the OGTT and BMI ≧̸33.0 kg/m^2^ was defined as obesity (21). Same as us to adopt self-reported pre-pregnancy BMI, Ijäs et al. (13) also showed that concomitant GDM further amplified the risk of macrosomia and CS in overweight and obese women (ppBMI ≧̸25 kg/m^2^). A prospective study in Spanish pregnant women demonstrated that overweight women (ppBMI ≧̸25 kg/m^2^), both with and without GDM, had significantly higher odds ratios for macrosomia, CS, and PIH, whereas non-overweight women with GDM did not (22); the diagnosis of GDM defined by the criteria of the National Diabetes Data Group (23) might have resulted in an underestimation of the differences between the groups. Aligned with them, we found that both women with maternal obesity alone (ppBMI ≧̸25 kg/m^2^) and women with maternal GAGM alone had higher adjusted odds of preeclampsia, CS, and macrosomia compared with non-obese women without GAGM, and women with the two combined conditions got worse outcomes than those with each condition alone in China. In accordance with our findings, a retrospective analysis involving 832 Chinese GDM women also showed that women who were obese before pregnancy had a 3.26-, 9.78-, and 8.04-fold risk to develop CS, gestational hypertension, and macrosomia than women with normal weight before pregnancy, and no risk difference of postpartum hemorrhage was observed (24); however, serious perinatal outcomes were not involved in their studies. Our present study added this evidence by comparisons of various adverse pregnancy outcomes and found that the combination of maternal pre-pregnancy obesity and GAGM rather than each alone could significantly enhance the risks of lower birth weight and fetal asphyxia when compared with normal weight women without GAGM. Maternal obesity was often in connection with hyperinsulinemia, insulin resistance, and rising inflammatory markers levels (25). Recent studies also reported that increasing maternal insulin resistance and inflammatory markers can independently predict adverse pregnancy outcomes (26, 27). These suggested that the combined maternal pre-pregnancy obesity and GAGM could amplify the risk of adverse perinatal outcomes with a multiplicative or additive effect and had worse impacts than each alone.

A few studies investigated the independent effects of maternal obesity and GAGM on the pregnancy outcome. The studies by Ijäs et al. (13) and Wahabi et al. (19) showed that compared with obesity, GDM had a modest effect on pregnancy outcomes, whereas Ricard et al. (22) and Hildén et al. (28) found that obesity had a greater independent effect on adverse outcomes compared to GDM. Nevertheless, the above conclusions were both obtained without comparing adverse outcomes between two groups and only used normal-weight women without GDM as the reference. In our present study, maternal obese non-GAGM women appeared to have worse outcomes than did maternal non-obese GAGM based on non-GAGM women without obesity as reference. However, taking the GAGM group as reference, the maternal pre-pregnancy obesity group only exhibited a remarkably higher risk of PIH, which indicated that elevated pre-gestational BMI was an independent risk factor for development of PIH (29, 30).

Most strikingly, the combination of pre-pregnancy obesity and GAGM not only was more strongly associated with each outcome but also had dramatically higher risks of adverse pregnancy outcomes than each one alone as reference in our study. Our results underlined the importance of pre-pregnancy and antenatal interventions especially for women with pre-pregnancy obesity. Moreover, adverse pregnancy outcomes caused by obesity and GAGM also constitute a huge global health and economic burden. Therefore, it is imperative to identify high-risk groups early and implement prevention and interventions to reduce the risk of adverse perinatal outcomes. Several works are worth doing. Firstly, it is important to find strategies such as medical lectures to help women of fertile age to know about adverse effects of obesity on the mother and the fetus during pregnancy, in order to promote better use of effective interventions. Secondly, further studies are needed to explore which lifestyle treatment options could best improve perinatal outcomes in obese women and whether there are some effective interventions to help reduce maternal weight before pregnancy, such as bariatric surgery. Thirdly, a series of effective strategies such as diet, physical exercise, weight management, and blood glucose self-monitoring should be followed by pregnant women during their pregnancy (31–34). For instance, Chen et al. (33) recently showed that internet combined with individualized exercise-based nursing intervention in GDM women can significantly reduce the high blood glucose levels and improve their pregnancy outcomes in relation to routine nursing intervention only. A randomized controlled trial by Bruno et al. (34) displayed that the adherence to a personalized, hypocaloric, low-glycemic, low-saturated fat diet started at the early pregnancy stage can prevent the occurrence of GDM in women with ppBMI ≧̸25 kg/m^2^.

Some limitations of our study need to be mentioned. Firstly, selection and information bias may exist as this was a retrospective cohort and single-center study. We will try to collect more data to make a multicenter and prospective study and further clarify the influences of maternal obesity and GAGM on pregnancy complications in our future study. Secondly, we only discussed the complications of mothers and offspring during the pregnancy period, and the long-term complications after delivery were not considered. Thirdly, the population in our study was Chinese residents, and whether our findings could be generalized to other populations still needs further study. Fourthly, the height and pre-pregnancy weight of our studied objects were self-reported at their first prenatal visit. Several studies have shown that self-reported maternal pre-pregnancy weight is well correlated with the actual weight (35, 36).

## Conclusions

Our study showed that maternal pre-pregnancy obesity and GAGM were independently associated with increased risks of adverse pregnancy outcomes. Furthermore, their combination had a greater impact on PIH, preeclampsia, CS, preterm birth, macrosomia, and total adverse maternal and neonatal outcomes than either obesity or GAGM alone in Chinese pregnant women. Our findings urged physicians to keep vigilant on the follow-up of pregnant women, especially those obese women with abnormal glucose tolerance. More effective intervention strategies are recommended to prevent serious adverse pregnancy outcomes among women with either obesity or GAGM.

## Data Availability Statement

The original contributions presented in the study are included in the article/supplementary material. Further inquiries can be directed to the corresponding authors.

## Ethics Statement

The studies involving human participants were reviewed and approved by the Human Research and Ethic Committee of Shanghai Jiao Tong University Affiliated Sixth People’s Hospital. The patients/participants provided their written informed consent to participate in this study.

## Author Contributions

J-BL and L-XL designed the study and reviewed and edited the manuscript. LM conducted the obstetric and surgical clinical practice. J-WW and Z-HZ collected the samples and clinical data. M-FL and J-FK worked together, performed the statistical analysis, and wrote the manuscript. All authors contributed to the article and approved the submitted version.

## Funding

The authors declare that this study received fundings from the National Key Research and Development Plan (2018YFC1314900 and 2018YFC1314905), the National Natural Science Foundation of China (grant numbers 81502316, 81170759, 81770813, and 82070866), the Translational Medicine National Key Science and Technology Infrastructure Open Project (grant number TMSK-2021-116), and the Exploratory Clinical Research Project of Shanghai Jiao Tong University Affiliated Sixth People’s Hospital (grant number ynts202105). The funders were not involved in the study design, collection, analysis, interpretation of data, the writing of this article, or the decision to submit it for publication.

## Conflict of Interest

The authors declare that the research was conducted in the absence of any commercial or financial relationships that could be construed as a potential conflict of interest.

## Publisher’s Note

All claims expressed in this article are solely those of the authors and do not necessarily represent those of their affiliated organizations, or those of the publisher, the editors and the reviewers. Any product that may be evaluated in this article, or claim that may be made by its manufacturer, is not guaranteed or endorsed by the publisher.
